# Defect identification of bare printed circuit boards based on Bayesian fusion of multi-scale features

**DOI:** 10.7717/peerj-cs.1900

**Published:** 2024-02-28

**Authors:** Xixi Han, Renpeng Li, Boqin Wang, Zhibo Lin

**Affiliations:** 1 School of Electronic Information, Zhongyuan University of Technology, Zhengzhou, Henan, China; 2 Anyang Iron and Steel Automation Software Co., Ltd, Zhengzhou, Henan, China

**Keywords:** Printed circuit board, Defect identification, Feature extraction, Migration learning

## Abstract

The aim of this article is to propose a defect identification method for bare printed circuit boards (PCB) based on multi-feature fusion. This article establishes a description method for various features of grayscale, texture, and deep semantics of bare PCB images. First, the multi-scale directional projection feature, the multi-scale grey scale co-occurrence matrix feature, and the multi-scale gradient directional information entropy feature of PCB were extracted to build the shallow features of defect images. Then, based on migration learning, the feature extraction network of the pre-trained Visual Geometry Group16 (VGG-16) convolutional neural network model was used to extract the deep semantic feature of the bare PCB images. A multi-feature fusion method based on principal component analysis and Bayesian theory was established. The shallow image feature was then fused with the deep semantic feature, which improved the ability of feature vectors to characterize defects. Finally, the feature vectors were input as feature sequences to support vector machines for training, which completed the classification and recognition of bare PCB defects. Experimental results show that the algorithm integrating deep features and multi-scale shallow features had a high recognition rate for bare PCB defects, with an accuracy rate of over 99%.

## Introduction

The printed circuit board (PCB) is an important carrier of electronic components and plays an important role in modern electronic equipment. The quality of PCBs is not only related to the reliability of electronic products but can also reflect the developmental level of a regional electronic industry. With the rapid upgrading and iteration of electronic technology, the demand for various types of electronic products continues to grow, leading to rapid growth of the PCB industry.

The quality of bare PCB determines whether electronic products can operate normally. During the production process of PCBs, it is difficult to avoid factors such as environmental interference, equipment aging, and manual operation errors. Each PCB production process may cause defects. Therefore, it is particularly important to conduct quality inspections and defect identification on bare PCBs during the PCB production process. An efficient bare PCB defect detection method can prevent defective bare PCBs from entering the next phase of the production process, which improves process efficiency, reduces the scrap rate of bare PCBs, and allows designers to analyze the defects for future improvements (Liu & Wen, 2021; Zhang, Jiang & Li, 2021). With the increasing accuracy and complexity of PCB design, the task of detecting and classifying defects has become even more challenging (Huang et al., 2020).

Machine vision technology is currently a research hotspot, and has great potential for application in the field of PCB defect detection (Kim et al., 2021). Machine vision technology uses image acquisition equipment to obtain visual information about objects and perform analysis and processing (Zhao et al., 2022; Sivaranjani & Senthilrani, 2023). It can achieve automated testing, improve production efficiency, and reduce production costs (Dong et al., 2022). Research on bare PCB defect detection algorithms based on machine vision theory can provide new ideas for machine vision technology, promote technological progress in related fields, and help expand the application field of machine vision and defect detection technology to areas such as medical (Coraci, Tognolo & Masiero, 2023; Gao et al., 2023), security (Mallaiyan Sathiaseelan et al., 2021; Ge, Dan & Li, 2020), and smart home technology (Yu & Pei, 2021; Talaat, Arafa & Metwally, 2020). The continuous improvement of this technology can also promote the cross-integration of machine vision and other disciplines, further promoting the improvement of industrial automation (Kshirsagar et al., 2022).

Image processing algorithms are key technologies in machine vision detection (Devi & Shitharth, 2021). Song, Kim & Park (2019) proposed a method based on a genetic algorithm to optimize the extraction of regions. This method extracts color features from solder joint images and then uses support vector machine (SVM) to classify solder joint defects. However, this proposed method is only applicable to defect detection of capacitors and resistors, and only uses component images obtained from RGB illumination for defect classification. Tsai & Huang (2018) proposed a global Fourier image reconstruction method based on traditional template matching techniques for detecting and locating small defects in non-periodic PCB images. This algorithm is insensitive to translation and lighting changes and can detect subtle defects one pixel wide in various non-periodic PCB images. It can also detect defects in the manufacturing environment. However, for very large images and images with high duplicate components, the detection efficiency and computational efficiency are not high. Li & Li (2017) proposed a bare PCB defect detection algorithm based on image edge features. The algorithm extracts gradient direction information entropy and the edge pixel density features of edge pixels, then combines SVM classifiers to achieve defect location. However, the calculation of neighborhood gradient direction information entropy is very sensitive to changes in gradient direction, and the defect feature extraction algorithm proposed in the article does not consider the impact of actual image distortion on this calculation. Lu et al. (2018) proposed a non-reference comparison framework for PCB defect detection that extracts the directional gradient histogram (HOG) and local binary patterns (LBP) features of PCB images, inputs them into SVM for supervised learning, and obtains two independent classification models. According to Bayesian fusion theory, the two models are then fused for defect classification. Lu et al. (2018) proved the effectiveness of fused features in defect classification problems, but their proposed algorithm cannot detect specific defect positions on the PCB surface. Jia & Liu (2022) improved the LeNet-5 network model for PCB character defect detection by changing the layer depth to examine the impact of different network architectures on model efficiency and adding a combination classifier in the fully connected layer to enhance the feature expression performance. However, this proposed method does not reduce the need for human intervention during the training process, and it is still a significant challenge to study the segmentation of overlapping character clusters. Shen, Liu & Sun (2020) established the “lightweight component category detection model” for lightweight PCB detection. This method enhanced the detection accuracy and achieved online monitoring of PCBs. Combined with a character recognition model, called component character, these two models were used for PCB component defect detection and recognition.

There are various types of defects in bare PCB boards, and most current algorithms only focus on a single feature in feature extraction, which makes it difficult to accurately describe the defects. Moreover, the dimension of feature extraction is usually too high. Defect detection methods based on deep learning rely on a large dataset of defects, and cannot guarantee the accuracy of detection for certain defects with smaller datasets. The key research focuses of the present study are feature extraction, feature reduction, and multi-feature fusion methods. This article proposes a bare PCB defect detection algorithm that combines multi-scale shallow features with deep features extracted by neural networks. Compared to single-scale features, multi-scale features have richer texture features and can better identify the detail information of an image. Convolutional neural network (CNN) can automatically learn features from datasets, allowing it to obtain new effective feature representations from new training data (Li, Kuo & Guo, 2020).

Building and training a deep learning network model requires a significant amount of time, expensive hardware, and a large number of labeled images. In the absence of a large number of datasets, transfer learning is a better solution (Hu, 2023). This article uses the VGGl6-Net network model trained on the ImageNet (Fang et al., 2021) dataset as the pre-trained model (Althubiti et al., 2022). The model was then fine-tuned, including adjusting network structure and parameters, to better fit the target dataset.

The first stage of this model is the extraction and fusion of defect features. To reduce the influence of background interference pixels on feature extraction, defect areas were segmented through image differentiation and morphological operations. Next, the multi-scale directional projection feature, the multi-scale gray-scale co-occurrence matrix feature, and the multi-scale gradient directional information entropy feature of the bare PCB defect image were extracted. Deep features were then extracted from defect images using a pre-trained VGG-16 convolutional neural network. Finally, using naive Bayesian theory, the feature information extracted by multiple algorithms was weighted and fused to construct a new feature vector.

The second stage of this model is defect classification and identification. The fused new feature vectors were used as training samples to train the support vector machine model and then complete the classification and recognition of bare PCB defects.

The rest of this the article is organized as follows: ‘Related work’ analyzes related work, including image preprocessing and feature extraction; ‘Method’ provides specific methods for feature extraction of defects and feature fusion; ‘Experimental result and discussion’ evaluates the performance of the proposed method and presents the defect detection accuracy and classification results using different algorithms; and ‘Conclusion’ summarizes the findings of this article and proposes future areas of research.

## Related Work

### mage preprocessing

**Figure 1:**
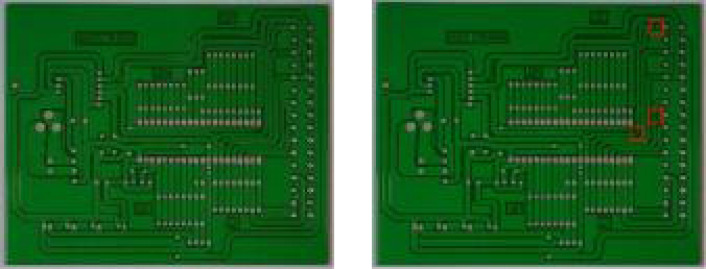
Image registration results.

The proposed model uses image enhancement and Gaussian filtering algorithms to preprocess the original image of the bare PCB to reduce the impact of lighting and noise on the image. Next, the PCB template images and PCB test images are registered using the Förstner corner detection algorithm (Naji et al., 2023). The pose correction and image registration of the image to be measured are completed through projection transformation. As shown in Fig. 1, the rectangular box represents the defect area obtained after calculating the difference between the template image and the test image.

**Figure 2:**
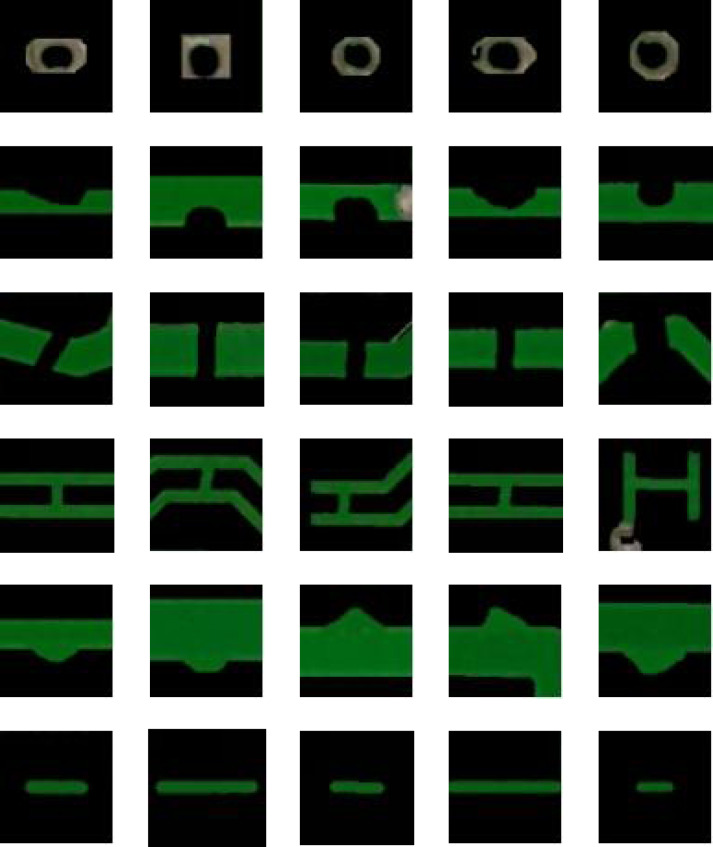
Examples of defect images.

The defect area of the image to be tested is then cropped on the bare PCB board. To reduce the impact of interfering pixels on feature extraction, the pixel value of the image area outside the defect area is set to 0. Some examples of defect images are shown in Fig. 2.

### Feature extraction

Image recognition is actually a classification process. To identify the category to which an image belongs, the image must be distinguished from images of different categories. Feature extraction requires the selected features of an image to both describe the image well and distinguish it from different types of images. In order to increase the gap between feature information classes and improve the classification performance of feature vectors for bare PCB defect images, this article proposes a defect extraction algorithm based on multi-scale feature fusion.

Figure 2 shows that the cropped bare PCB defect image makes it difficult to extract rich feature information at a single resolution. Therefore, the proposed model performs down-sampling on the defect images. Based on the Gaussian pyramid, a multi-scale defect image is constructed. The image resolution is gradually reduced with the down-sampling, and the information is gradually enriched. Four down-sampling operations are performed on each image, 1–4 layers of pyramid images are constructed, and the features from each scale image are extracted, separately.

**Figure 3:**
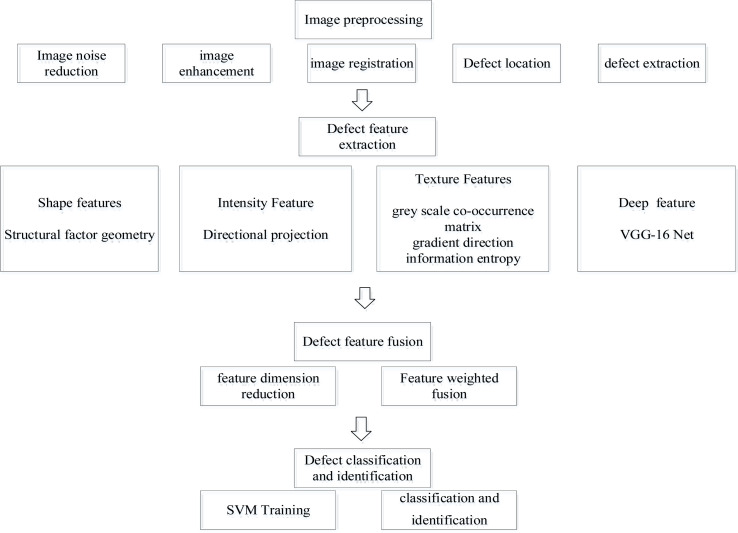
Technology roadmap.

The technology roadmap of this model is shown in Fig. 3.

## Method

### Multi-scale grey scale co-occurrence matrix feature extraction

The grey scale co-occurrence matrix features are extracted in the 0° direction of each layer pyramid image. This model uses the following statistics as features of the grey scale co-occurrence matrix:

Width represents the width of the co-occurrence matrix; *c*_*ij*_ represents the term in the co-occurrence matrix.

Angular second moment (Asm) refers to the sum of squares of the values of the elements in the grayscale co-occurrence matrix, which reflects the uniformity of the image grayscale distribution and the texture thickness:

(1) \begin{eqnarray*}\mathrm{Asm}=\sum _{i,j=0}^{\text{width}}{c}_{ij}^{2}\end{eqnarray*}

Correlation (Cor) measures the similarity of elements in the spatial gray level co-occurrence matrix in the row or column direction:

(2) \begin{eqnarray*}\mathrm{Cor}= \frac{\sum _{i,j=0}^{\text{width}} \left( i-{u}_{x} \right) \left( j-{u}_{y} \right) {c}_{ij}}{{s}_{x}{s}_{y}} \end{eqnarray*}

where,

(3) \begin{eqnarray*}{u}_{x}=\sum _{i,j=0}^{\text{width}}i\cdot {c}_{ij}\end{eqnarray*}

(4) \begin{eqnarray*}{u}_{y}=\sum _{i,j=0}^{\text{width}}j\cdot {c}_{ij}\end{eqnarray*}

(5) \begin{eqnarray*}{s}_{x}^{2}=\sum _{i,j=0}^{\text{width}}{ \left( i-{u}_{x} \right) }^{2}{c}_{ij}\end{eqnarray*}

(6) \begin{eqnarray*}{s}_{y}^{2}=\sum _{i,j=0}^{\text{width}}{ \left( i-{u}_{y} \right) }^{2}{c}_{ij}\end{eqnarray*}

Homogeneity (Hom) measures the number of local changes in image texture:

(7) \begin{eqnarray*}\mathrm{Hom}=\sum _{i,j=0}^{\text{width}} \frac{1}{1+(i-j)^{2}} {c}_{ij}\end{eqnarray*}

Contrast (Con) reflects the clarity of the image and the depth of the texture grooves:

(8) \begin{eqnarray*}\mathrm{Con}=\sum _{i,j=0}^{\text{width}}(i-j)^{2}{c}_{ij}\end{eqnarray*}

The grey scale co-occurrence matrix features of the 1–4 layers of pyramid images are calculated separately. The multi-scale grey scale co-occurrence matrix feature vectors are obtained by concatenating the feature vectors.

### Multi-scale directional projection feature extraction

**Figure 4:**
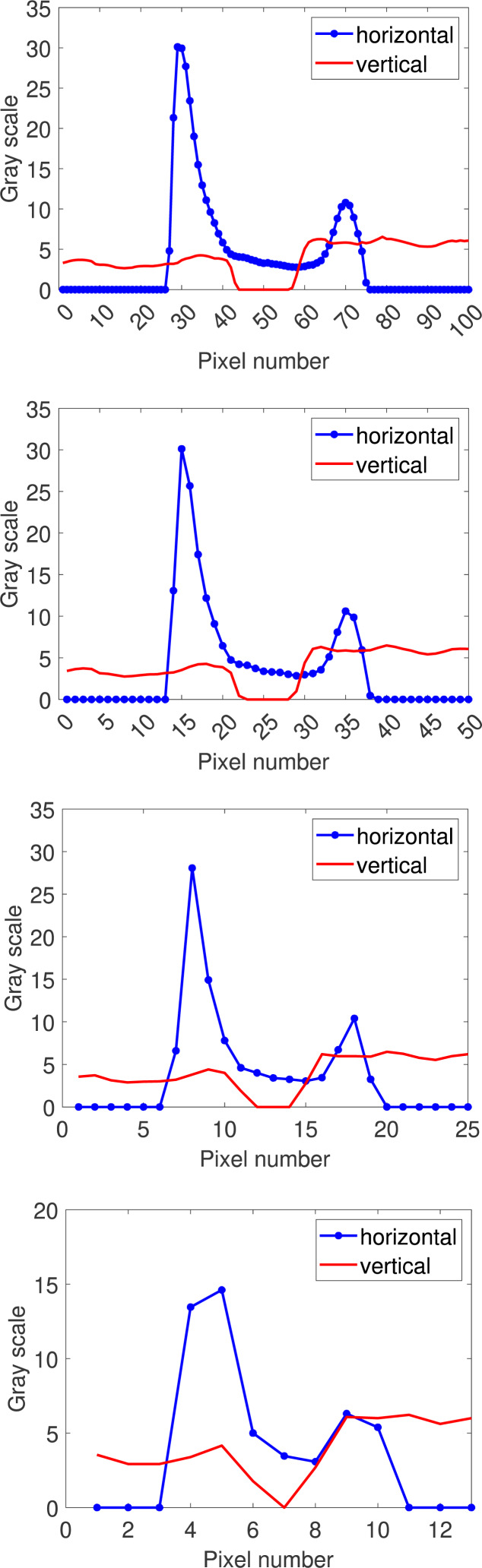
Multi-scale directional projection function for open circuit images.

Directional projection refers to the horizontal and vertical projection of regional grayscale values. The calculation formula is as follows:

(9) \begin{eqnarray*}{P}_{\text{Vert}} \left( c \right) = \frac{1}{n \left( c+{c}^{{^{\prime}}} \right) } \sum _{ \left( r+{r}^{{^{\prime}}},c+{c}^{{^{\prime}}} \right) \in R}I \left( r+{r}^{{^{\prime}}},c+{c}^{{^{\prime}}} \right) \end{eqnarray*}

(10) \begin{eqnarray*}{P}_{\mathrm{Hor}} \left( r \right) = \frac{1}{n \left( r+{r}^{{^{\prime}}} \right) } \sum _{ \left( r+{r}^{{^{\prime}}},c+{c}^{{^{\prime}}} \right) \in R}I \left( r+{r}^{{^{\prime}}},c+{c}^{{^{\prime}}} \right) \end{eqnarray*}

where *R* refers to region and represents the target area; *I* refers to image, which represents the image where the region is located; $ \left( {r}^{{^{\prime}}},{c}^{{^{\prime}}} \right) $ represents the top left corner of the bounding rectangle parallel to the smallest axis in the input area; $n \left( x \right) $ represents the number of region points in the corresponding row (*r* + *r*′) or column (*c* + *c*′). Horizontal projection returns a one-dimensional function that reflects changes in vertical grayscale values. Similarly, vertical projection returns a function that reflects changes in horizontal grayscale values. Horizontal and vertical projection functions for 1–4 layers of pyramid images are then drawn separately, using open circuit defects as an example, as shown in Fig. 4.

The directional projection function cannot effectively characterize the shape features of defects. The directional projection function is also directly input into the classifier as a feature vector, which may cause data redundancy. The known one-dimensional curve can be regarded as a one-dimensional signal in time-domain space. Therefore, this model extracts the time-domain features of the multi-scale directional projection functions of each defect image as feature vectors. The time-domain feature representation method is as follows:

Root mean square:

(11) \begin{eqnarray*}{X}_{\mathrm{RMS}}=\sqrt{ \frac{1}{N} \sum _{i=1}^{N}{g}_{i}^{2}}\end{eqnarray*}

Root amplitude:

(12) \begin{eqnarray*}{X}_{r}={ \left[ \frac{1}{N} \sum _{i=1}^{N}\sqrt{ \left\vert {g}_{i} \right\vert } \right] }^{2}\end{eqnarray*}

Skewness:

(13) \begin{eqnarray*}{X}_{\mathrm{sk}}= \frac{1}{N} \sum _{i=1}^{N}{g}_{i}^{3}\end{eqnarray*}

Average amplitude:

(14) \begin{eqnarray*}{X}_{\text{mean}}= \frac{1}{N} \sum _{i=1}^{N} \left\vert {g}_{i} \right\vert \end{eqnarray*}

Peak value:

(15) \begin{eqnarray*}{X}_{\text{peak}}=\max \nolimits \left\vert {g}_{i} \right\vert \end{eqnarray*}

Kurtosis:

(16) \begin{eqnarray*}{K}_{u}= \frac{ \frac{1}{N} \sum _{i=1}^{N}{ \left( {g}_{i}-\overline{g} \right) }^{4}}{{X}_{\mathrm{RMS}}^{4}} \end{eqnarray*}

Waveform factor:

(17) \begin{eqnarray*}{S}_{F}= \frac{{X}_{\mathrm{RMS}}}{{X}_{\text{mean}}} \end{eqnarray*}

Pulse factor:

(18) \begin{eqnarray*}{I}_{F}= \frac{{X}_{\text{peak}}}{{X}_{\text{mean}}} \end{eqnarray*}

Margin:

(19) \begin{eqnarray*}L= \frac{{X}_{\text{peak}}}{{X}_{r}} \end{eqnarray*}

Crest factor:

(20) \begin{eqnarray*}{I}_{\mathrm{CF}}= \frac{{X}_{\text{peak}}}{{X}_{\mathrm{RMS}}} \end{eqnarray*}

where *N* represents the number of pixel sequences, g_*i*_ represents the directional projection function value corresponding to the *i*th pixel sequence. The time-domain characteristics of the directional projection function of the 1–4 layers of pyramid images are then calculated and the time-domain features are concatenated to obtain the multi-scale directional projection feature vectors of the image.

### Multi-scale gradient direction information entropy feature extraction

#### Gray information entropy

Entropy refers to the degree of chaos in a system, which is used to measure the uncertainty of information. The smaller the entropy, the more certain the information, so entropy also reflects the amount of information. Assuming there is a discrete random variable *X* with a probability distribution function of *p*(*x*), its entropy *H*(*X*) can be represented by the following equation:

(21) \begin{eqnarray*}H(X)=-\sum _{x\in \chi }p(x)\log \nolimits p(x)\end{eqnarray*}

Image entropy reflects the average amount of information in an image, and describes the number and frequency of occurrences of grayscale values based on the analysis and statistics of the obtained image grayscale values. The one-dimensional grayscale entropy of a grayscale image can be represented by the following equation:

(22) \begin{eqnarray*}H=-\sum _{i=0}^{255}{P}_{i}\log \nolimits {P}_{i}\end{eqnarray*}

Based on one-dimensional entropy, this model uses a feature quantity that can reflect the spatial characteristics of the gray distribution to form the two-dimensional entropy of an image:

(23) \begin{eqnarray*}{P}_{ij}=f(i,j)/{N}^{2}\end{eqnarray*}

where *f*(*i*, *j*) is the frequency of the occurrence of feature binary (*i*, *j*) and *N* isthe size of the image. The definition Eq. (24) represents the two-dimensional entropy of discrete images:

(24) \begin{eqnarray*}H=\sum _{i=0}^{255}{P}_{ij}\log \nolimits {P}_{ij}\end{eqnarray*}

This equation can reflect the grayscale value at a certain pixel position and the comprehensive characteristics of the gray distribution of its surrounding pixels.

#### Image gradient

The image gradient represents the axis and partial derivative of the current pixel point to the axis, and can also be understood as the change speed of the pixel gray value. In mathematics, the Sobel mask operator is used to calculate the approximate gradient of changes in the horizontal and vertical directions of an image. The following common operators can be used, with *A* defined as the source image, and *G*_*x*_ and *G*_*y*_ defined as the approximate horizontal and vertical gradients of an image, respectively. The calculation method is as follows:

(25) \begin{eqnarray*}{G}_{x}= \left[ \begin{array}{@{}lll@{}} \displaystyle -1&\displaystyle 0&\displaystyle 1\\ \displaystyle -2&\displaystyle 0&\displaystyle 2\\ \displaystyle -1&\displaystyle 0&\displaystyle 1 \end{array} \right] \ast A, {G}_{y}= \left[ \begin{array}{@{}lll@{}} \displaystyle 1&\displaystyle 2&\displaystyle 1\\ \displaystyle 0&\displaystyle 0&\displaystyle 0\\ \displaystyle -1&\displaystyle -2&\displaystyle -1 \end{array} \right] \ast A\end{eqnarray*}

where ∗ represents the convolution operation between the operator and the image.

**Figure 5:**
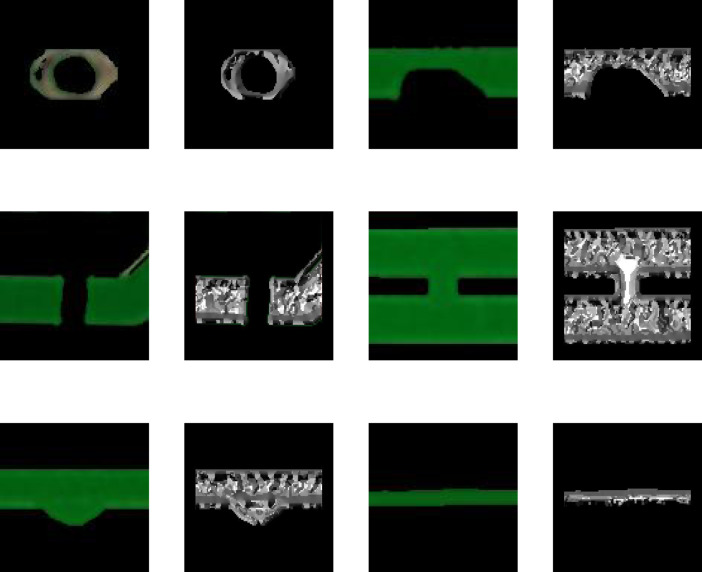
Gradient direction feature map.

The gradient direction feature is reflected in the image in the form of grayscale values. First, the original image is converted to a grayscale image, and then the pixels under the mask are calculated according to the formula. The obtained results replace the values of pixels at the center of the mask, and after calculating all pixels, the original image gradient direction feature map is obtained. One image was selected for each type of defect for experimentation, and the obtained gradient direction feature map is shown in Fig. 5.

This model calculates the grayscale information entropy of the gradient direction feature map of bare PCB defects based on the two-dimensional grayscale entropy calculation method of the image. Then, based on the multi-scale image construction method studied in the previous section, the gradient direction information entropy is calculated for the 1–4 layers of pyramid images. The obtained results are then serialized as multi-scale gradient direction information entropy features for bare PCB defects.

### Deep semantic feature extraction based on transfer learning

To further explore the deep semantic features of bare PCB defect images, the model proposed in this article uses a convolutional neural network to extract features from the datasets. The VGG-16 network, proposed by Simonyan & Zisserman (2014) was selected as the pre-training model. The VGG-16 model consists of a convolutional layer, a pooling layer, and a fully connected layer. The network model structure is shown in Table 1.

**Table 1:** VGG-l6 network structure.

Number of layers	Model	Kernel configuration		
		Size	Quantity	Step size
1	Convolution	3 × 3	64	1
2	Convolution	3 × 3	64	1
3	Max-pooling	2 × 2	–	2
4	Convolution	3 × 3	128	1
5	Convolution	3 × 3	128	1
6	Max-pooling	2 × 2	–	2
7	Convolution	3 × 3	256	1
8	Convolution	3 × 3	256	1
9	Convolution	3 × 3	256	1
10	Max-pooling	2 × 2	–	2
11	Convolution	3 × 3	512	1
12	Convolution	3 × 3	512	1
13	Convolution	3 × 3	512	1
14	Max-pooling	2 × 2	–	2
15	Convolution	3 × 3	512	1
16	Convolution	3 × 3	512	1
17	Convolution	3 × 3	512	1
18	Max-pooling	2 × 2	–	2
19	Full-connection	4096	–	–
20	Full-connection	4096	–	–
21	Full-connection	1000	–	–
22	SoftMax	–	–	–

Each convolutional layer applies a series of convolutional checks to the input data through convolutional operations for feature extraction, generating a feature mapping set. The maximum pooling layer aggregates input data through sliding windows to reduce feature mapping. Pooling operations can also improve the ability of features to describe images. The fully connected layer is used to generate feature vectors. After each layer, there is a nonlinear layer, ReLU, represented as *f*(*x*) = max(0, *x*), used to achieve rapid convergence of network training.

In the process of transfer learning, there are two models for fine-tuning. The first method is to freeze all convolutional feature extraction layers of the pre-trained model and only perform fine-tuning training operations on the classification layer; the second method is to fine tune all convolutional feature extraction and classification layers of the pre-trained model (Lamsiyah et al., 2023).

This model uses the first fine-tuning method. The known convolutional layer feature maps are output in the form of matrix sets. If directly converted into a one-dimensional vector, the dimensionality of the vector is very large, leading to overfitting problems and affecting classification efficiency (Bai & Tang, 2018). In order to reduce the risk of overfitting, the number of parameters and computational complexity are reduced. This model removes the last five layers of the VGGl6-Net network model and replaces them with a global average pooling layer and a fully connected layer with 512 nodes.

**Figure 6:**
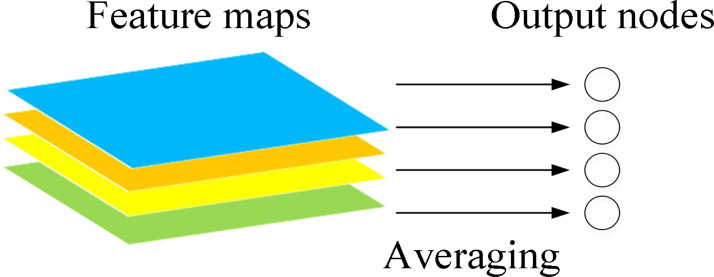
Schematic diagram of global pooling layer.

The function of global average pooling is to add all the pixel values of the feature map and find a balance to obtain a numerical value. By using this numerical value to represent the corresponding feature map, the spatial parameters are reduced, and the robustness of the network is improved (Hsiao et al., 2019). The schematic diagram is shown in Fig. 6.

**Figure 7:**
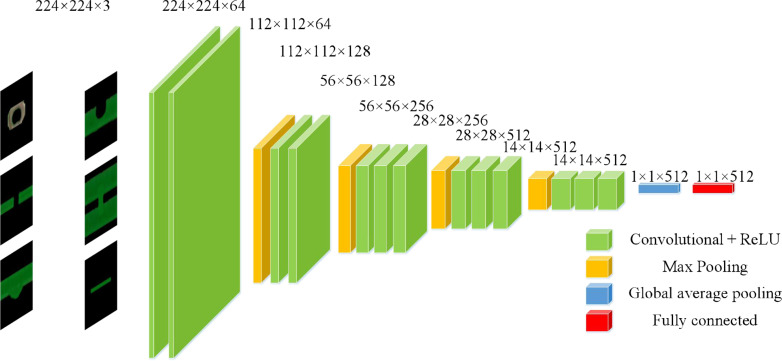
The VGG-l6 model after fine tuning.

The feature maps output from the global average pooling layer are used as inputs to the fully connected layer, and the 512-dimensional feature vectors outputted from the fully connected layer are used as the deep features of the bare PCB defect images. The structure of the adjusted VGGl6 net model is shown in Fig. 7.

### Feature fusion

The different semantic features extracted from the same bare PCB defect image reflect different image information. This model integrates multi-scale grayscale co-occurrence matrix features, multi-scale directional projection features, multi-scale gradient directional information entropy features, and deep semantic features extracted by the VGG-l6 neural network to increase the diversity of feature information. If each group of feature vectors were simply added or subtracted, it would cause problems, such as high dimensionality of feature vectors and high computational complexity, so it is necessary to reduce the dimensionality of the feature vectors. It is also necessary to assign different weights to different feature vectors because different feature vectors have different scale ranges and classification performance, and direct addition and subtraction would ignore the imbalance of the data.

This model uses principal components analysis (PCA) to reduce the dimensionality of the extracted feature vectors (Jiang & Sun, 2023), and the high contribution features are extracted as effective features for bare PCB defect images.

Assuming there are M samples $ \left\{ {X}_{1},{X}_{2,},\ldots ,{X}_{M} \right\} $ andeach sample has an N-dimensional feature vector ${X}_{i}={ \left( {x}_{1}^{i},{x}_{2}^{i},\ldots ,{x}_{N}^{i} \right) }^{T}$, then each feature *x*_*j*_ has its own characteristic value.

The steps for the PCA are as follows:

**Step 1:** All features are centralized using demeaning.

Each feature is averaged and then its own mean is subtracted.

(26) \begin{eqnarray*}\overline{{x}_{n}}= \frac{1}{M} \sum _{i=1}^{M}{x}_{n}^{i}\end{eqnarray*}

**Step 2:** The Covariance matrix *C*, is calculated taking features A and S as examples.

(27) \begin{eqnarray*}C= \left[ \begin{array}{@{}ll@{}} \displaystyle \mathrm{cov} \left( {x}_{1},{x}_{1} \right) &\displaystyle \mathrm{cov} \left( {x}_{1},{x}_{2} \right) \\ \displaystyle \mathrm{cov} \left( {x}_{2},{x}_{1} \right) &\displaystyle \mathrm{cov} \left( {x}_{2},{x}_{2} \right) \end{array} \right] .\end{eqnarray*}

In the above matrix, the variances of features *x*_1_ and *x*_2_ are on the diagonal, and the covariance is found on the non-diagonal. A covariance greater than 0 indicates that if one of *x*_1_ and *x*_2_ *x*_2_ increases, the other also increases; a covariance less than 0 means when one increases, the other decreases; when the covariance is 0, the two are independent. The larger the absolute value of covariance, the greater the impact the two have on each other. The solution formula for $\mathrm{cov} \left( {x}_{1},{x}_{1} \right) $ is shown in Eq. (28), and the same applies to the others.

(28) \begin{eqnarray*}\mathrm{cov} \left( {x}_{1},{x}_{1} \right) = \frac{\sum _{i=1}^{M} \left( {x}_{1}^{i}-\overline{{x}_{1}} \right) \left( {x}_{1}^{i}-\overline{{x}_{1}} \right) }{M-1} \end{eqnarray*}

The covariance matrix *C* of M samples under N-dimensional features are obtained in this covariance calculation formula.

**Step 3:** The eigenvalues of the covariance matrix are and the corresponding eigenvectors are identified.

The eigenvalue *λ* of the Covariance matrix *C* and the corresponding eigenvector µ(each eigenvalue corresponds to a eigenvector) is calculated with the following formula:

(29) \begin{eqnarray*}C\mu =\lambda \mu .\end{eqnarray*}

There are N eigenvalues *λ*, and each *λ*_*i*_ corresponds to a feature vector *μ*_*i*_. The feature values are sorted in descending order, with the largest k selected, producing a set of $ \left\{ \left( {\lambda }_{1},{\mu }_{1} \right) , \left( {\lambda }_{2},{\mu }_{2} \right) ,\ldots , \left( {\lambda }_{k},{\mu }_{k} \right) \right\} $.

**Step 4:** The original features are projected onto the selected feature vector to obtain the new K-dimensional features after dimensionality reduction.

Dimensionality reduction is performed by selecting and projecting the largest eigenvalues and corresponding eigenvectors. For each sample *X*^*i*^, the original feature is ${ \left( {x}_{1}^{i},{x}_{2}^{i},\ldots ,{x}_{n}^{i} \right) }^{T}$, and the projected new feature is ${ \left( {y}_{1}^{i},{y}_{2}^{i},\ldots ,{y}_{k}^{i} \right) }^{T}$. The new feature is calculated using Eq. (30):

(30) \begin{eqnarray*} \left[ \begin{array}{@{}l@{}} \displaystyle \begin{array}{@{}l@{}} \displaystyle {y}_{1}^{i}\\ \displaystyle {y}_{2}^{i} \end{array}\\ \displaystyle \cdot \\ \displaystyle \cdot \\ \displaystyle \begin{array}{@{}l@{}} \displaystyle \cdot \\ \displaystyle {y}_{k}^{i} \end{array} \end{array} \right] = \left[ \begin{array}{@{}l@{}} \displaystyle \begin{array}{@{}l@{}} \displaystyle \begin{array}{@{}l@{}} \displaystyle {\mu }_{1}^{T}{ \left( {x}_{1}^{i},{x}_{2}^{i},\ldots ,{x}_{n}^{i} \right) }^{T}\\ \displaystyle {\mu }_{2}^{T}{ \left( {x}_{1}^{i},{x}_{2}^{i},\ldots ,{x}_{n}^{i} \right) }^{T} \end{array}\\ \displaystyle \cdot \end{array}\\ \displaystyle \cdot \\ \displaystyle \cdot \\ \displaystyle {\mu }_{k}^{T}{ \left( {x}_{1}^{i},{x}_{2}^{i},\ldots ,{x}_{n}^{i} \right) }^{T} \end{array} \right] \end{eqnarray*}

so that each sample *X*^*i*^ is changed from the original ${X}^{i}={ \left( {x}_{1}^{i},{x}_{2}^{i} \right) }^{T}$ to the current ${X}^{i}={y}_{1}^{i}$.

This model selects appropriate features by determining the amount of information contained in the feature values. According to Eq. (30), the new feature vectors calculated by principal component analysis are arranged in descending order. The size of the eigenvalues corresponds to the amount of information contained in the eigenvalues, which also indicates the contribution rate of the principal components. This model selects the feature values with a cumulative contribution rate of over 99.8% and ranking first as the fusion feature values. After the principal component calculation, the first six principal components of the multi-scale gray level co-occurrence matrix feature vector, the first seven principal components of the multi-scale directional projection feature vector, the first two principal components of the multi-scale gradient directional information entropy feature vector, and the first 68 principal components of the deep feature vector were used as the dimensionality reduced feature vectors for feature fusion.

The naive Bayesian theory is then used to calculate the weights of each group of feature vectors (Shitharth et al., 2022). The posterior probability of each group of eigenvectors is calculated through the dataset, and then their respective weight values are calculated. The dataset is first divided and then the conditional probability and prior probability of each category are calculated according to the training set. The posterior probability of the eigenvector is then calculated using the Bayesian formula. The maximum posterior probability is used as the final posterior probability of the eigenvector (Krishna & Divya, 2022).

The specific steps to calculate the posterior probability of eigenvectors and assign weights to each group of eigenvectors are as follows:

**Step 1:** Assuming there is a feature vector $x= \left\{ {x}_{1},{x}_{2},\ldots ,{x}_{n} \right\} $ and a category set *C* = *c*_1_, *c*_2_, …, *c*_*k*_, then the posterior probability calculation formula of the Naive Bayes classifier is as follows:

(31) \begin{eqnarray*}P({c}_{i}{|}x)= \frac{P({c}_{i})P(x{|}{c}_{i})}{P(x)} \end{eqnarray*}

where *P*(*c*_*i*_) represents the prior probability of the category in the training set, *P*(*x*|*c*_*i*_) represents the conditional probability of eigenvector *x* under category *c*_*i*_, and *P*(*x*) represents the marginal probability of feature vector *x*. According to the naive Bayesian theory, denominator a is a constant in classification and can be omitted, so the posterior probability can be simplified as *P*(*c*_*i*_|*x*) ∝ *P*(*c*_*i*_)*P*(*x*|*c*_*i*_).

**Step 2:** The prior probability *P*(*c*_*i*_) and conditional probability *P*(*x*|*c*_*i*_) are calculated. The prior probability *P*(*c*_*i*_) represents the frequency of occurrence of category *c*_*i*_ in the training set, and the calculation method is as follows:

(32) \begin{eqnarray*}P \left( {c}_{i} \right) = \frac{ \left\vert {D}_{{c}_{i}} \right\vert }{ \left\vert D \right\vert } \end{eqnarray*}

where |*D*_*c*_*i*__| represents the number of samples belonging to category *c*_*i*_ in the training set and |*D*| represents the total number of samples in the training set. The calculation of conditional probability *P*(*x*|*c*_*i*_) is based on the naive Bayesian classifier assumption, also called the feature conditional independence assumption. According to this assumption, conditional probability can be defined as the product of the conditional probability of each feature, as shown in the following formula:

(33) \begin{eqnarray*}P(x{|}{c}_{i})=\prod _{j=1}^{n}P({x}_{j}{|}{c}_{i})\end{eqnarray*}

where *x*_*j*_ represents the *j*th feature of feature vector *x* and *P*(*x*_*j*_|*c*_*i*_) represents the conditional probability of the occurrence of feature *x*_*j*_ under category *c*_*i*_. For discrete features, *P*(*x*_*j*_|*c*_*i*_) can be directly defined as the frequency of feature *x*_*j*_ appearing under category *c*_*i*_, and the calculation formula is as follows:

(34) \begin{eqnarray*}P({x}_{j}{|}{c}_{i})= \frac{{|}{D}_{{c}_{i},{x}_{j}}{|}}{{|}{D}_{{c}_{i}}{|}} \end{eqnarray*}

where |*D*_*c*_*i*_,*x*_*j*__| represents the number of samples in the training set that belong to category *c*_*i*_ and feature *x*_*j*_ appears, and |*D*_*c*_*i*__| represents the number of samples belonging to category *c*_*i*_ in the training set. For continuous features, assuming that they conform to the Gaussian distribution, the conditional probability *P*(*x*_*j*_|*c*_*i*_) can be calculated using the probability density function of the Gaussian distribution, as shown in the following formula:

(35) \begin{eqnarray*}P({x}_{j}{|}{c}_{i})= \frac{1}{\sqrt{2\pi {\sigma }_{{c}_{i},j}^{2}}} \exp \nolimits \left( - \frac{({x}_{j}-{\mu }_{{c}_{i},j})^{2}}{2{\sigma }_{{c}_{i},j}^{2}} \right) \end{eqnarray*}

where *μ*_*c*_*i*_,*j*_ and *σ*_*c*_*i*_,*j*_ represent the mean and standard deviation of feature *x*_*j*_ under category *c*_*i*_, respectively, as calculated from the training set.

**Step 3:** The posterior probability of the feature vector is calculated according to the naive Bayesian classifier, as follows:

(36) \begin{eqnarray*}P({c}_{i}{|}x)\propto P({c}_{i})\prod _{j=1}^{n}P({x}_{j}{|}{c}_{i})\end{eqnarray*}

where *P*(*c*_*i*_) represents the prior probability of category *c*_*i*_ in the training set and *P*(*x*_*j*_|*c*_*i*_) represents the conditional probability of the occurrence of feature *x*_*j*_ under category *c*_*i*_. Then the maximum a posteriori estimation is the final posterior probability of the eigenvector, and is calculated as follows:

(37) \begin{eqnarray*}{P}_{\mathrm{max}}({c}_{i}{|}x)={\mathrm{argmax}}_{{c}_{i}\in C}P({c}_{i})\prod _{j=1}^{n}P({x}_{j}{|}{c}_{i})\end{eqnarray*}

**Step 4:** The weight coefficients *w*_*i*_ of each group of feature vectors, defined as the normalization result of the maximum a posteriori estimation, are calculated as follows:

(38) \begin{eqnarray*}{w}_{i}= \frac{{P}_{\mathrm{max}}({c}_{i}{|}x)}{\sum _{i=1}^{n}{P}_{\mathrm{max}}({c}_{i}{|}x)} \end{eqnarray*}

Due to the presence of four sets of feature vectors in this model, *n* = 4.

After feature dimensionality reduction and weight allocation, the feature vectors need to be connected together to complete feature fusion. This model uses parallel fusion as the feature vector stitching method. Assuming there is a bare PCB defect image sample *I*, after dimensionality reduction and weighting, $A= \left\{ {A}_{1},{A}_{2},\ldots ,{A}_{n} \right\} $ and $B= \left\{ {B}_{1},{B}_{2},\ldots ,{B}_{n} \right\} $ are obtained, and the feature vector after parallel fusion is $V= \left\{ {C}_{1},{C}_{2},\ldots ,{C}_{n} \right\} $, where *C*_*i*_ is defined as the following equation:

(39) \begin{eqnarray*}{C}_{i}=\sqrt{({A}_{i})^{2}+({B}_{i})^{2}}\end{eqnarray*}

If the number in digits of feature vectors A and B is not equal, then 0 is added to the low dimensional feature vectors.

Before dimensionality reduction, the parallel fusion dimension of feature vectors had a total of 512 dimensions. After dimensionality reduction, the parallel fusion dimension of feature vectors was reduced to 68 dimensions. The feature dimensionality was reduced significantly, and the noise was eliminated. Different weight coefficients were assigned to each group of feature vectors based on different classification performance; the important features were strengthened and the non important features were weakened.

The pseudocode of the proposed algorithm is shown in Table 2.

**Table 2:** The pseudocode of the proposed algorithm.

**Algorithm** Multi-feature fusion based on principal component analysis and Bayesian theory
1:**Intput:** Image *I* of PCB bare board defects to be detected
2:Construct 4-layer Gaussian pyramid image $ \left\{ {I}_{1},{I}_{2,},{I}_{3},{I}_{4} \right\} $ of defect image I of PCB bare
board to be detected
3:Calculate the multi-scale gray level co-occurrence matrix feature vector
$\text{GLCM}= \left[ {\text{GLCM}}_{1},{\text{GLCM}}_{2},{\text{GLCM}}_{3},{\text{GLCM}}_{4} \right] $ of *I* via Eqs. (1)–(8)
4:Calculate the multi-scale directional projection feature vectors
$\mathrm{pro}= \left[ {X}_{\mathrm{RMS}},{X}_{r},{X}_{\mathrm{sk}},{X}_{\text{mean}},{X}_{\text{peak}},{K}_{u},{S}_{F},{I}_{F},L,{I}_{\mathrm{CF}} \right] $ for *I* via Eqs. (9)–(20)
5:Calculate the multi-scale gradient direction information entropy feature vector
Ent = [*H*_1_, *H*_2_, *H*_3_, *H*_4_] of *I* for *I* via Eqs. (21)–(25)
6:Calculating the deep semantic feature vector *D* = [*d*_1_, *d*_2_, *d*_3_, …, *d*_*n*_], *n* = 512 of *I* based
on the improved VGG-16 neural network
7:Extract features from image *I* to obtain M feature vectors $ \left\{ {X}_{1},{X}_{2,},\ldots ,{X}_{M} \right\} $
8:Reduce the dimension of $ \left\{ {X}_{1},{X}_{2,},\ldots ,{X}_{M} \right\} $ via Eqs. (26)–(30), obtain N feature vectors
$ \left\{ {X}_{1},{X}_{2,},\ldots ,{X}_{n} \right\} $
9:Calculate the weight $ \left\{ {W}_{1},{W}_{2,},\ldots ,{W}_{n} \right\} $ of each feature vector of $ \left\{ {X}_{1},{X}_{2,},\ldots ,{X}_{N} \right\} $ via Eqs. (31)–(38)
10:Calculate the feature vector *V* after parallel weighted fusion via Eq. (39)
11:**Output:** the feature vector $V= \left\{ {C}_{1},{C}_{2},\ldots ,{C}_{n} \right\} $ after parallel weighted fusion

## Experiment Results and Discussion

The Open Laboratory of Intelligent Robotics at Peking University has a bare PCB dataset with various defects (leaks, mouse bites, open circuits, short circuits, burrs, and residual copper) (Huang et al., 2020), which can be used for image detection, classification, and registration tasks. Each type of defect is distributed on 10 different bare PCB images, with a total of 690 defect images in the database. Each image has 2–6 defective areas. After defect extraction, the sample size statistics are shown in Table 3.

**Table 3:** Statistics of defect sample quantity.

Defect type	Number of images/piece	Number of defect samples/piece
Leaks	115	442
Mouse bites	115	442
Open circuits	116	419
Short circuits	116	499
Burrs	114	424
Residual copper	114	434
Total	690	2660

This experiment used the following hardware: an Intel(R) Core(TM) i5-8300H CPU @ 2.30 GHz 2.30 GHz with a GTX 1050Ti graphics card. Bare PCB defect detection requires machine vision algorithms that can be developed quickly and efficiently, as well as a stable operating environment. This article proposes a newly-designed and developed bare PCB defect detection algorithm based on the Halcon platform. Halcon is a commercial software with powerful machine vision capabilities and high scalability, which supports multiple programming languages and development environments.

The experiments were conducted on the Halcon and Matlab platforms. First, based on the feature extraction method outlined previously, the multi-scale gray level co-occurrence matrix feature vector $\text{GLCM}= \left[ {\text{GLCM}}_{1},{\text{GLCM}}_{2},{\text{GLCM}}_{3},{\text{GLCM}}_{4} \right] $ of 1∼4 layers of Gaussian pyramid image were extracted for each bare PCB defect image. The feature vector of gray level co-occurrence matrix of the first layer Gaussian pyramid image was ${\text{GLCM}}_{1}= \left[ {\mathrm{Asm}}_{1},{\mathrm{Cor}}_{1},{\mathrm{Hom}}_{1},{\mathrm{Con}}_{1} \right] $, the feature vector of gray level co-occurrence matrix of the second layer Gaussian pyramid image was ${\text{GLCM}}_{2}= \left[ {\mathrm{Asm}}_{2},{\mathrm{Cor}}_{2},{\mathrm{Hom}}_{2},{\mathrm{Con}}_{2} \right] $, the feature vector of gray level co-occurrence matrix of the third layer Gaussian pyramid image was ${\text{GLCM}}_{3}= \left[ {\mathrm{Asm}}_{3},{\mathrm{Cor}}_{3},{\mathrm{Hom}}_{3},{\mathrm{Con}}_{3} \right] $, and the feature vector of gray level co-occurrence matrix of the fourth layer Gaussian pyramid image was ${\text{GLCM}}_{4}= \left[ {\mathrm{Asm}}_{4},{\mathrm{Cor}}_{4},{\mathrm{Hom}}_{4},{\mathrm{Con}}_{4} \right] $. The multi-scale gradient direction information entropy eigenvector of 1∼4 layers of Gaussian pyramid image was Ent = [*H*_1_, *H*_2_, *H*_3_, *H*_4_], and the multi-scale directional projection feature vector was $\mathrm{pro}= \left[ {X}_{\mathrm{RMS}},{X}_{r},{X}_{\mathrm{sk}},{X}_{\text{mean}},{X}_{\text{peak}},{K}_{u},{S}_{F},{I}_{F},L,{I}_{\mathrm{CF}} \right] $. Fused in parallel with the deep semantic feature vector *D* = [*d*_1_, *d*_2_, *d*_3_, …, *d*_*n*_], *n* = 512 extracted from the convolutional neural network to form a new feature vector. Then, the new feature vector was used as input to SVM, and the SVM classifier model was trained to classify bare PCB defect images.

The dataset was divided as follows: 70% of the dataset was used as the training set, 10% as the validation set, and 20% as the testing set. To verify the superiority of the multi-feature fusion algorithm in image description compared to a single feature, the extracted multi-scale gray level co-occurrence matrix feature vector, multi-scale gradient direction information entropy feature vector, multi-scale direction projection feature vector, and deep feature vector were respectively input into the SVM classifier for classification and recognition.

The confusion matrix obtained based on deep feature recognition is shown in Table 4.

**Table 4:** Deep feature confusion matrix.

	Leaks	Mouse bites	Open circuits	Short circuits	Burrs	Residual copper	FP
Leaks	88	0	1	2	0	0	3
Mouse bites	0	78	15	0	0	0	15
Open circuits	0	0	68	0	0	0	0
Short circuits	0	2	0	95	0	0	2
Burrs	0	8	0	1	80	0	9
Residual copper	0	0	0	0	4	87	4
FN	0	10	16	3	4	0	33

The confusion matrix obtained based on multi-scale gradient direction information entropy feature is shown in Table 5.

**Table 5:** Multi-scale gradient direction information entropy feature confusion matrix.

	Leaks	Mouse bites	Open circuits	Short circuits	Burrs	Residual copper	FP
Leaks	88	0	1	8	3	1	13
Mouse bites	0	75	9	12	1	0	22
Open circuits	0	11	72	7	3	1	22
Short circuits	0	1	2	68	4	0	7
Burrs	0	1	0	3	73	0	4
Residual copper	0	0	0	0	0	85	0
FN	0	13	12	30	11	2	68

The confusion matrix obtained based on multi-scale gray co-occurrence matrix feature is shown in Table 6.

**Table 6:** Multi-scale gray co-occurrence matrix feature confusion matrix.

	Leaks	Mouse bites	Open circuits	Short circuits	Burrs	Residual copper	FP
Leaks	66	0	0	0	0	0	0
Mouse bites	0	82	1	0	0	0	1
Open circuits	0	0	63	0	0	0	0
Short circuits	22	5	20	98	48	6	101
Burrs	0	1	0	0	36	0	1
Residual copper	0	0	0	0	0	81	0
FN	22	6	21	0	48	6	103

The confusion matrix obtained based on multi-scale directional projection feature is shown in Table 7.

**Table 7:** Multi-scale directional projection feature confusion matrix.

	Leaks	Mouse bites	Open circuits	Short circuits	Burrs	Residual copper	FP
Leaks	80	6	0	1	0	0	7
Mouse bites	0	81	11	1	0	0	12
Open circuits	5	0	68	10	0	0	15
Short circuits	0	1	0	80	8	0	9
Burrs	3	0	5	3	76	3	14
Residual copper	0	0	0	3	0	84	3
FN	8	7	16	18	8	3	60

The analysis results of different confusion matrices show that fused features can better describe image detail features compared to single features, with a significant improvement in accuracy.

To verify the classification effect of the SVM fusion multi-feature classification algorithm on bare PCB defect images, the algorithm proposed in this article was compared with the CNN algorithm using AlexNet (Kavitha & Rao, 2019), VGG-16, and ResNet-50 as the selected CNN network models. Accuracy (acc), precision (pre), recall, and F1 score were used as evaluation indicators for classification performance and were calculated, as follows:

(40) \begin{eqnarray*}\mathrm{acc} \left( A,B \right) = \frac{A}{B} \end{eqnarray*}

(41) \begin{eqnarray*}\mathrm{pre}= \frac{\mathrm{TP}}{\mathrm{TP}+\mathrm{FP}} \end{eqnarray*}

(42) \begin{eqnarray*}\text{recall} \left( {A}_{i},{B}_{i} \right) = \frac{{A}_{i}}{{B}_{i}} \end{eqnarray*}

(43) \begin{eqnarray*}F1= \frac{2\cdot \mathrm{pre}\cdot \text{recall}}{\mathrm{pre}+\text{recall}} \end{eqnarray*}

where, *A* represents the sum of the correct number of sample discrimination, *B*represents the total number of samples, *B*_*i*_ represents the number of samples of each type, TP represents the number of correctly identified samples in the category, FP represents the number of cost class samples misjudged by other class samples, and FNrepresents the number of samples in this category that were mistakenly classified as other categories.

The comparative classification results are shown in Table 8.

**Table 8:** Comparative classification results.

Algorithm	Evaluating indicator	Leaks	Mouse bites	Open circuits	Short circuits	Burrs	Residual copper	Average value
Algorithm in this article	Accuracy Rate/%	99.43						
	Precision Rate/%	100	100	98.82	100	97.67	100	99.42
	Recall rate/%	100	97.73	100	98.98	100	100	99.45
	F1 score/%	100	98.85	99.41	99.49	98.82	100	99.43
AlexNet	Accuracy Rate/%	97.16						
	Precision Rate/%	92.63	97.67	100	96.04	97.65	100	97.33
	Recall rate/%	100	95.45	95.24	98.98	98.81	94.25	97.12
	F1 score/%	96.17	96.55	97.56	97.49	98.22	97.04	97.17
VGG-16	Accuracy Rate/%	98.11						
	Precision Rate/%	100	98.84	98.75	96.00	95.45	100	98.17
	Recall rate/%	100	96.59	94.05	97.96	100	100	98.10
	F1 score/%	100	97.7	96.34	96.97	97.67	100	98.11
ResNet-50	Accuracy Rate/%	98.68						
	Precision Rate/%	100	96.67	100	97.00	100	98.86	98.76
	Recall rate/%	100	98.86	96.43	98.98	97.62	100	98.65
	F1 score/%	100	97.75	98.18	97.98	98.8	99.43	98.69

**Figure 8:**
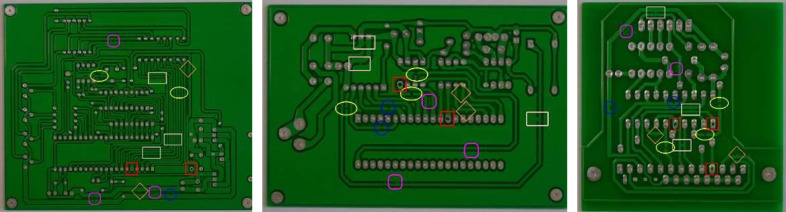
Defect identification results.

Figure 8 shows the detection and recognition results of defects in the tested images of different types of bare PCB boards using the algorithm proposed in this article. The rectangle in the figure represents a short circuit, the ellipse represents an open circuit, the diamond represents a burr, the square represents a leak, the circle represents a mouse bite, and the square with rounded corners represents residual copper. The comparative experimental results (Table 8) shows that, compared to traditional CNN classification algorithms, the SVM classification algorithm that integrates multi-scale shallow features and deep semantic features had a significant improvement in the accuracy of bare PCB defect image classification, demonstrating that the feature extraction algorithm proposed in this article can obtain more effective information about defects.

## Conclusion

Aiming at the problems existing in the field of bare PCB defect detection, this article proposes a multi-scale feature extraction method based on Gaussian pyramid that extracts multi-scale gray level co-occurrence matrix features, multi-scale directional projection features, and multi-scale gradient direction information entropy features, and uses a convolutional neural network to further extract image deep semantic features to enrich feature information. This article establishes a feature fusion method based on principal component analysis and Bayesian theory. First, based on principal component analysis, the extracted feature vectors are dimensionally reduced, greatly reducing both feature dimension and computational complexity. Then, Bayesian theory is used to calculate the weight values of different feature vectors, and multiple sets of vectors are fused in parallel. Compared to a single feature, fused features have stronger image representation capabilities. In addition, SVM has unique advantages in handling small sample problems. The proposed model fuses all the extracted features into a new feature vector to enhance the ability of the features to describe the image and converts it into a feature sequence, which is input into SVM for training to achieve bare PCB defect classification and recognition. The experimental results prove that the proposed algorithm can quickly and accurately identify leaks, mouse bites, open circuits, short circuits, burrs, and residual copper defects, with an accuracy rate of over 99% and a certain degree of stability.

The research in this article provides some ideas for bare PCB detection, but improvements are needed in future work. This article only focuses on some specific issues of bare PCB defect detection, and the proposed method is not yet suitable for all complex production environments. The interference mode, interference type, and interference degree in actual production environments cannot be predicted, such as lens interference, electromagnetic interference, or light source interference, which were not considered in the research process of this article. Therefore, improving the anti-interference ability of PCB defect detection algorithms in complex noise environments has significant practical significance. In addition, the defect detection algorithm proposed in this article can detect bare PCB boards in production and quickly detect defects that occur during the production process, but cannot evaluate the severity of defects. Future research should include measuring the size of different defects and evaluating the impact of defects on the quality of bare PCBs to better improve the production process.

## Supplemental Information

10.7717/peerj-cs.1900/supp-1 Supplemental Information 1 Code

## Additional Information and Declarations

### Data Availability

The following information was supplied regarding data availability: The code is available in the Supplementary File and Zenodo: Han, X. (2023). Code [Data set]. Zenodo. https://doi.org/10.5281/zenodo.10068752
